# An Audit and Quality Improvement Project Regarding the Management of Patients With Eating Disorders Within the West Glasgow CAMHS Team

**DOI:** 10.1192/bjo.2022.96

**Published:** 2022-06-20

**Authors:** Sukhmeet Singh, Maryjane Roberts, Alexandra Clark, Christabel Boyle

**Affiliations:** NHS Greater Glasgow and Clyde, Glasgow, United Kingdom

## Abstract

**Aims:**

There has been a significant increase in presentations of people with eating disorders (ED) within CAMHS in relation to the pandemic with a significant pressure on services to continue to provide evidence-based treatments for an expanding number of severely unwell patients. The first aim was to assess the quality of referrals received for patients with suspected ED and to then implement an intervention regarding the way that referrals are handled. The second aim was to establish a process for handling and monitoring patients already open to the service.

**Methods:**

An initial and repeat survey was sent to staff within the team. The survey included the Mental Health Professional Stress Scale (MHPSS). An audit was conducted to establish the quality of referrals from GPs based on the Junior Marsipan guidelines. Data were collected on physical measures and the written content of referrals for March 2020–21 and March 2021–22.

Duty clinicians were asked to screen referrals and prompt GPs to submit recordings of physical parameters for the referrals to be triaged. In addition, a weekly meeting in a “board round” format was implemented to discuss new referrals and 40–50 existing patients each week depending on risk. We developed a physical health monitoring clinic once per week.

**Results:**

MHPSS scores remained high between initial and follow-up surveys, with slightly increased mean scores for workload, organisational structure and processes, and lack of resources. Referrals from 2020–2021 (N = 26) and those from 2021–2022 (N = 39) were screened. The majority had a diagnosis of anorexia nervosa. Most referrals had records of height and weight (73.1 to 82.1%). 53.8% of referrals in the re-audit period required prompting for physical recordings to be submitted. There was no change in the written content of referrals at re-audit, with only 46.2% recording risk, 51.3% recording estimated onset and 56.4% documenting body image.

There was a slight reduction in the mean time between referral and diagnosis from 44.1 to 34.2 days. The weekly board round received positive feedback (N = 10) with 70–100% answering agree/strongly agree to statements such as manage patients’ care safely, obtaining urgent advice and physical monitoring.

**Conclusion:**

The processes summarised above have been successful in improving the efficiency surrounding the management of patients with ED. Unfortunately, there has been no improvement in the stress levels of staff; we hope to conduct a focus group to better understand this. A referral proforma should be developed by the wider service for GPs to complete.

